# Estimating the Epicenter of a Future Strong Earthquake in Southern California, Mexico, and Central America by Means of Natural Time Analysis and Earthquake Nowcasting

**DOI:** 10.3390/e23121658

**Published:** 2021-12-09

**Authors:** Jennifer Perez-Oregon, Panayiotis K. Varotsos, Efthimios S. Skordas, Nicholas V. Sarlis

**Affiliations:** 1 Departamento de Física, Escuela Superior de Física y Matemáticas, Instituto Politécnico Nacional, UP Zacatenco C.P., Mexico City 07738, Mexico; jnnfr.po@gmail.com; 2 Solid Earth Physics Institute, Department of Physics, National and Kapodistrian University of Athens, Panepistimiopolis Zografos, 157 84 Athens, Greece; eskordas@phys.uoa.gr; 3 Section of Geophysics and Geothermy, Department of Geology and Geoenvironment, National and Kapodistrian University of Athens, Panepistimiopolis Zografos, 157 84 Athens, Greece; panvar@noa.gr; 4 Section of Condensed Matter Physics, Department of Physics, National and Kapodistrian University of Athens, Panepistimiopolis Zografos, 157 84 Athens, Greece

**Keywords:** natural time analysis, order parameter fluctuations, earthquake nowcasting, Mexico, Central America

## Abstract

It has recently been shown in the Eastern Mediterranean that by combining natural time analysis of seismicity with earthquake networks based on similar activity patterns and earthquake nowcasting, an estimate of the epicenter location of a future strong earthquake can be obtained. This is based on the construction of average earthquake potential score maps. Here, we propose a method of obtaining such estimates for a highly seismically active area that includes Southern California, Mexico and part of Central America, i.e., the area N${}_{10}^{35}$W${}_{80}^{120}$. The study includes 28 strong earthquakes of magnitude M ≥7.0 that occurred during the time period from 1989 to 2020. The results indicate that there is a strong correlation between the epicenter of a future strong earthquake and the average earthquake potential score maps. Moreover, the method is also applied to the very recent 7 September 2021 Guerrero, Mexico, M7 earthquake as well as to the 22 September 2021 Jiquilillo, Nicaragua, M6.5 earthquake with successful results. We also show that in 28 out of the 29 strong M ≥7.0 EQs studied, their epicenters lie close to an estimated zone covering only 8.5% of the total area.

## 1. Introduction

**Figure 1:**
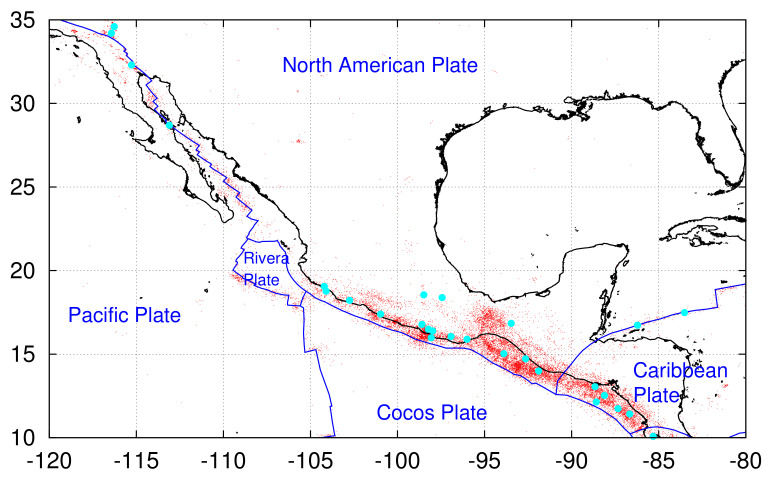
Map of the study area N${}_{10}^{35}$W${}_{80}^{120}$ together with the plate boundaries (blue) according to Bird [73]. The epicenters of the 28 strong EQs with M≥7.0 during 1989 to 2020 are shown with cyan bullets while those of the EQs with M≥4.0 by red dots.

Earthquakes (EQs) in Mexico and the surrounding region of Southern California and Central America are very common and extremely strong, see, e.g., References [1,2,3,4] and references therein. When focusing in the region N${}_{10}^{35}$W${}_{80}^{120}$ (shown in Figure 1), more than twenty-eight EQs with a magnitude M ≥7.0 have occurred there since 1989. In the present paper, we employ the natural time analysis (NTA) [5,6,7,8,9,10] and earthquake nowcasting (EN) [11,12,13,14,15,16,17,18] aiming at forecasting the epicenter of such a strong future EQ.

Recently, our group has combined [19] NTA and EN together with the properties of the earthquake networks based on similar activity patterns (ENBOSAP) [20,21] for a similar purpose in the Eastern Mediterranean region where seismicity is much less intense than in the region N${}_{10}^{35}$W${}_{80}^{120}$ of our present interest. We see that when strong EQs are frequent, as is the present case, we can simplify the approach of Reference [19] just by using NTA and EN.

Natural time was introduced [5] in 2001 as a general method to analyze time-series resulting from complex systems [7]. It has been applied to a variety of fields, such as condensed matter physics [22,23,24], geophysics [6,25,26,27,28,29,30,31,32,33,34], civil engineering [35,36,37,38], climatology [39,40,41,42], and biomedical engineering [43,44]. Within the concept of NTA, it has been shown that the variance $\kappa_{1}$ of natural time χ may be considered as an order parameter for seismicity [3,45,46,47,48,49] as well as in acoustic emission before fracture [28,50] or in other self-organized critical phenomena such as ricepiles [51] and avalanches in the Olami–Feder–Christensen [52] earthquake model [53] or in the Burridge–Knopoff [54] train model [55]. Especially for seismicity, the study of this order parameter by means of its variability [56] revealed [9,21,57,58,59,60,61,62,63] the presence of characteristic minima before the occurrence of strong EQs. Interestingly, the precursory Seismic Electric Signals (SES) activities [64,65], which are a series of low frequency electric signals (f≤ 1 Hz) observed before strong EQs [64,65,66,67,68,69,70] have been shown to appear [19,71,72] almost simultaneously with the variability minima.

Earthquake nowcasting has been introduced by Rundle et al. [11] and allows the evaluation of the current state of seismic hazard for strong EQs by the number of smaller EQs that occur in the time interval between two strong ones. It has been applied for the estimation of seismic risk to global megacities [12,15] as well as of the risk of great earthquakes that may generate mega-tsunamis [74]. EN has also been applied for the estimation of induced seismicity [75,76] and offers unique possibilities for the estimation of the seismic risk worldwide through global sources of seismic catalogs, see, e.g., References [77,78,79,80], cf. local EQ catalogs have also been used, such as the one from the Institute of Geodynamics of the National Observatory of Athens [81,82,83] for EN in Greece (Chouliaras, personal comm. 2019).

## 2. Materials and Methods

### 2.1. EQ Data and the Tectonics of the Study Area

The area of interest here is Southern California, Mexico, and Central America, see Figure 1. We used the United States National Earthquake Information Center (NEIC) PDE catalog—these data are available from the United States Geological Survey (USGS), cf. [84]—in the region N${}_{10}^{35}$W${}_{80}^{120}$ and considered all EQs recorded during the period of almost 50 years from 1 January 1973 to 21 September 2021 with magnitude $M\geq M_{thres}=4.0$.

The interaction of the continental block with the oceanic provinces that surround Mexico has resulted in the current geographic layout. Mexico is located in a region where five tectonic plates: North America, Pacific, Rivera, Cocos, and Caribbean, are in regular interaction. The major fault zones, the spreading zones, and the subduction zones determine the boundaries between these plates [85]. The North America’s displacement is to the southwest, the Eastern Pacific’s to the northwest, the Cocos and Rivera’s to the northeast, and the Caribbean’s to the east; this disposition enhances the likelihood of a large seismic occurrence. Mostly, the interplay that occurs on the Mexican Pacific’s southern coast, where the Cocos plate subducts beneath the North American plate, is the primary source of earthquakes in this country.

Oceanic plates are considered to be substantially more rigid than continental plates due to their olivine-rich composition. The Pacific Plate, the largest of the Earth’s tectonic plates, is extensively an oceanic plate. The plate border deformation zones in the continental crust are, without a doubt, far larger (tens to hundreds of kilometers) than the normally narrow (10 km) boundaries seen in oceanic plates [86]. In the Pacific region, the Baja California Peninsula is moving northwest, separating from the rest of the continent; the oceanic plate of the Cocos is being assimilated by the continent in the southern Pacific of Mexico, from Cabo Corrientes in the state of Jalisco to Central America; this subduction occurs along an oceanic trench known as the Acapulco or Mesoamerican megashear [87]. Further, in the seismic regions of the Gulf of Mexico and the Caribbean there are geological forces of cortical separation (also referred as tension or distensive) operating on the continental limits, and as a result of the movement of the continental tectonic plates of North America towards the west and the Caribbean towards the east, they advance on the deepest bottoms of the oceanic basins. In the Pacific Plate, in southern California and in Baja California, the plates are migrating northwesterly relative to the North American plate along a series of transformation faults (San Andreas fault) connecting the extension centers, whose activity is gradually separating this territory from the rest of the continent, for which it will become an island in approximately 10 million years. Similarly, oceanic faults allow magma to escape, generating an expansion of the ocean floor [88,89].

The Rivera microplate is located in southern Baja California, right at the gateway to the Sea of Cortez where the magnetic lineaments of the ocean floor indicate how the gap between the Pacific plate and the Rivera plate, positioned between fracture zones, grows. Due to the movement that the Cocos and Rivera plates have towards the northeast of the Mexican Republic, a portion of these plates dips under the North American plate, causing great earthquakes to occur along the coast of Jalisco, Colima, Michoacán, Guerrero, Oaxaca, and Chiapas; yet we cannot determine if these large earthquakes were caused by the Cocos or Rivera plate movement. The Cocos plate is built in the Eastern Pacific mountain range, that goes from the Rivera fault zone to the Galapagos mountain chain. It is located off the coastlines of Michoacán, Guerrero, Oaxaca, and Chiapas, and it dips into the continental crust, leading to a displacement that causes earthquakes throughout the Pacific coast.

Cocos plate subducts beneath the North American plate at a rate of about 12 cm/yr from 20 Ma to 11 Ma and 6 cm/yr from 11 Ma to present [90]. Along the central portion of the Middle American Trench, the subduction interface shows a significant variety in along strike dip angles; also, the middle section of the plate, near Acapulco, has a horizontal slab. The trace/strike of the Neogene volcanic arc, which trends at a 17 degree angle to the trace/strike of the trench, demonstrates the along strike change in the dip. The dip is 50 degrees to the northwest near the Rivera Plate junction, and 30 degrees to the southeast near the Tehuantepec Ridge [91]. The slab in central Mexico has returned to its current location near the southern boundary of the Neogene volcanic arc, as shown by the southward movement of the volcanic arc [92].

A triple point to the southeast of the Tehuantepec Ridge divides the North American plate from the Caribbean plate, and the Cocos plate begins to subduct under it; this poses substantial natural hazards to most of central and southern Mexico. The Yucatan Peninsula, for its part, is rotating clockwise, and the Trans-Mexican Volcanic Belt is still active [93].

### 2.2. Natural Time Analysis Background: An Order Parameter for Seismicity and Its Minima

**Figure 2:**
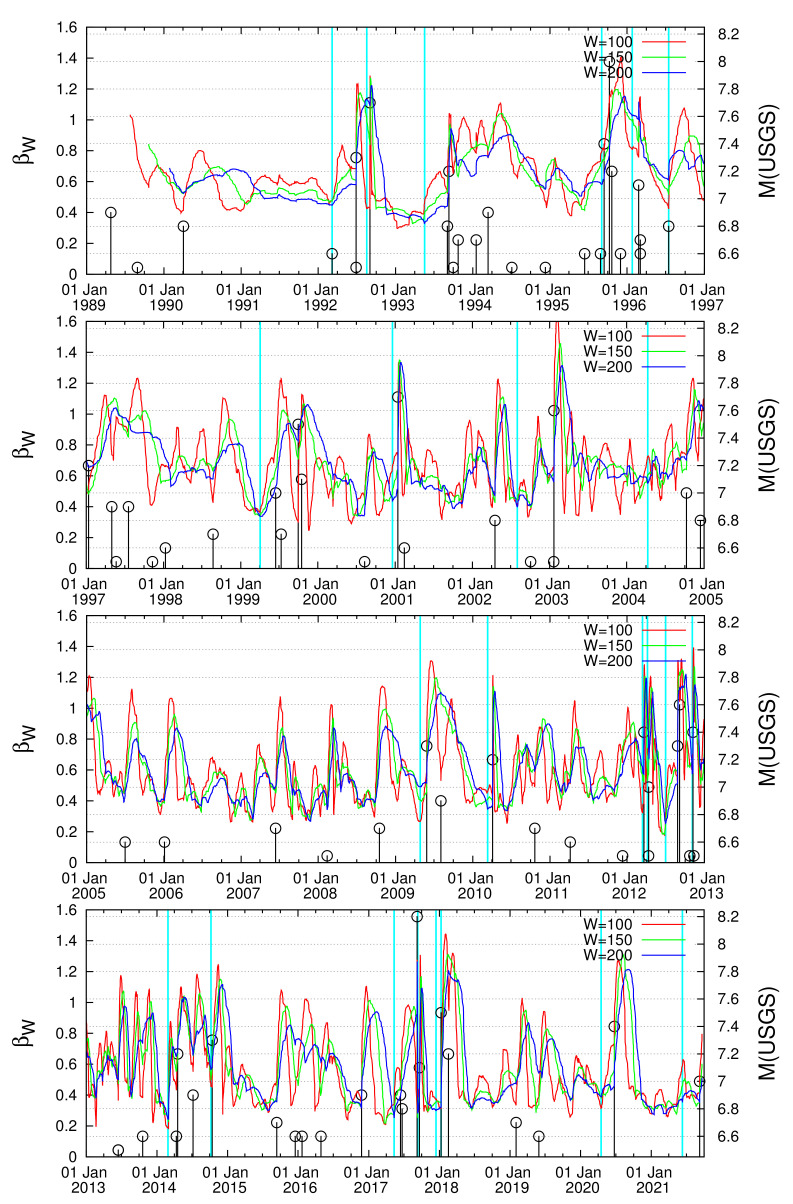
The variabilities $\beta_{W}$ for W = 100, 150, and 200 consecutive EQs versus the conventional time for the period 1989 to 21 September 2021. The EQ magnitudes (right scale) are depicted by the (black) vertical lines ending in circles. The cyan vertical lines indicate the dates at which minima of $\beta_{W}$ have been observed and have been selected for drawing the 〈EPS〉 maps.

**Figure 3:**
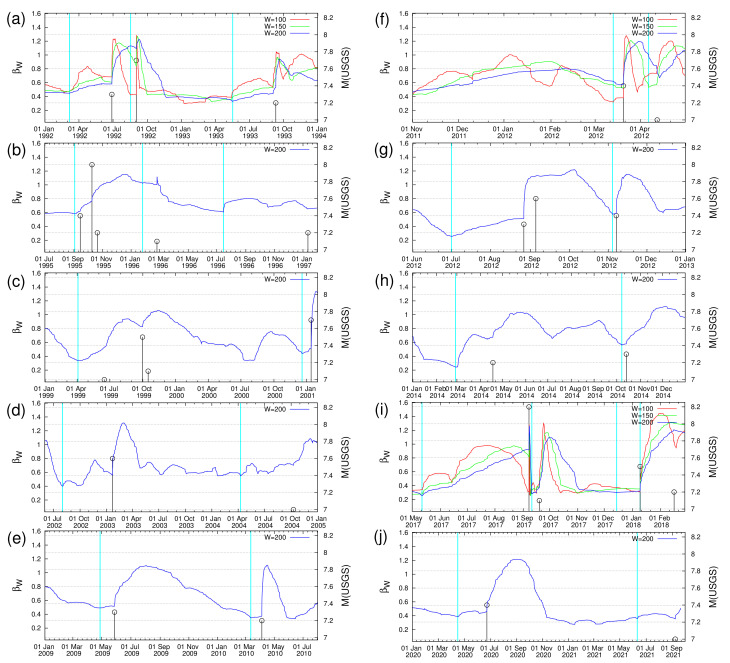
Excerpts of Figure 2 in expanded time scale. Each excerpt (**a**)-(**j**) corresponds to a different time period, in which strong M≥7.0 EQs occurred: (**a**) 1992-1993, (**b**) 1995-1997, (**c**) 1999-2001, (**d**) 2003-2004, (**e**) 2009-2010, (**f**) March and April 2012, (**g**) August, September, and November 2012, (**h**) 2014, (**i**) 2017-2018, and (**j**) 2020-2021. In each panel, the variability $\beta_{200}$ is drawn while $\beta_{150}$ and $\beta_{100}$ are drawn only in the cases in which a $\beta_{W}$ minimum is identified based on W= 150 or 100 (see the text and Table 1).

The natural time $\chi_{k}$ for the occurrence of the *k*-th EQ of the energy $Q_{k}$ in a time series comprising *N* EQs is defined as $\chi_{k}=k/N$. Hence, the evolution of the pair $\left(\chi_{k},p_{k}\right)$ is studied in the NTA, where

(1) $$p_{k}=\frac{Q_{k}}{\sum_{n=1}^{N}Q_{n}}$$

is the normalized energy and $Q_{k}$ is estimated by means of the relation [94] $Q_{k}\propto 10^{1.5M_{k}}$, where $M_{k}$ stands for the EQ magnitude. The variance $\kappa_{1}=\langle \chi^{2}\rangle -{\langle \chi \rangle }^{2}$ of natural time χ weighted for $p_{k}$, namely

(2) $$\kappa_{1}=\sum_{k=1}^{N}p_{k}{\left(\chi_{k}\right)}^{2}-{\left(\sum_{k=1}^{N}p_{k}\chi_{k}\right)}^{2},$$

can be considered as an order parameter for seismicity [45]. The fluctuations of this order parameter of seismicity in an EQ catalog can be studied by using a fixed-length sliding natural time window containing a number *W* of consecutive EQs by means of the procedure described in References [9,95]. The window length *W* is selected to correspond to the average number of EQs that occur within the crucial scale [96] of a few months, or so, which is the average lead time of SES activities. To this end, we estimate all the $\kappa_{1}$ values from the subexcerpts of consecutive 6 to *W* EQs within the excerpt of the EQ catalog of *W* EQs and use them for the calculation of their average value $\mu_{W}\left(\kappa_{1}\right)$ and standard deviation $\sigma_{W}\left(\kappa_{1}\right)$. The variability of the order parameter of seismicity $\kappa_{1}$ is given by [7,56]

(3) $$\beta_{W}\equiv \frac{\sigma {}_{W}\left(\kappa_{1}\right)}{\mu {}_{W}\left(\kappa_{1}\right)},$$

while its temporal evolution can then be pursued by sliding the natural time window of *W* consecutive EQs, event by event through the EQ catalog, see Figure 2 and Figure 3. In such a procedure, we assign to $\beta_{W}$ the occurrence time of the EQ which follows the last event of the excerpt of *W* EQs in the catalog.

### 2.3. Earthquake Nowcasting and Earthquake Potential Score

**Figure 4:**
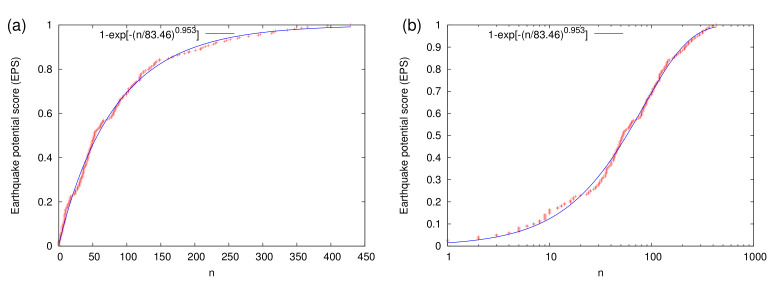
The empirical cumulative distribution function (red plus symbols) of the number *n* of EQs with M ≥4.0 that occur between two EQs of magnitude M ≥6.0 in the study area N${}_{10}^{35}$W${}_{80}^{120}$ of Figure 1 during the period 1989 to 2020. This equals to the EPS according to EQ nowcasting and has been calculated on the basis of 218 EQ cycles. The corresponding Weibull model fit [79] is also shown with the blue curve in lin–lin (**a**) and lin–log (**b**) diagram.

Rundle et al. [11] proposed EQ nowcasting as a method for estimating the seismic risk through the current state of fault systems in the progress of the EQ cycle identified on the basis of natural time (cf. more recently the construction of time series resembling the EQ cycle has been extensively studied in References [16,18,97]). To estimate the seismic risk, EN uses an EQ catalog to calculate from the number of ‘small’ EQs, defined as those with magnitude $M<M_{\lambda }$ but above a threshold $M_{\sigma }$, i.e., $M\in [M_{\sigma },M_{\lambda })$, the level of hazard for ‘large’ $M\geq M_{\lambda }$ EQs. As mentioned in the Introduction, the EQ catalogs adopted [11,12,19,77,78,79,80,98] are global seismic catalogs such as the Advanced National Seismic System Composite Catalog or the NEIC PDE catalog and for $M_{\sigma }$ the completeness threshold of the EQ catalog is usually selected [11]. Along these lines, the magnitude threshold $M_{\sigma }=4.0$ has been considered [12,78] for applications in areas such as Greece, Japan, and India that lie outside the United States. In EN, one employs the natural time concept and counts the number *n* of ‘small’ EQs that occur after a ‘large’ EQ—*n* stands for the waiting natural time or interoccurrence natural time. Then, the current number n(t) of the ‘small’ EQs since the last occurrence of a ‘large’ one is compared to the cumulative distribution function (CDF) of the interoccurrence natural time Prob[n<n(t)]. To estimate Prob[n<n(t)], it should be ensured [11] that we have enough data to span at least 20 or more ‘large’ EQ cycles. In EN, the EQ potential score (EPS) equals the CDF value,

(4) EPS=Prob[n<n(t)],

and measures the level of the current hazard, see Figure 4a. In References [11,12,77,78,79] the seismic risk for various cities of the world was estimated through the following procedure: After calculating the CDF Prob[n<n(t)] within a large area, the number $\tilde{n}$ of the small EQs around a city, i.e., those occurring within a circular region of epicentral distances r<R since the occurrence of the last ‘large’ EQ in this circular region, is found. Given that EQs exhibit ergodicity—see, e.g., References [99,100,101]—Rundle et al. [12] suggested that the seismic risk around a city can be estimated by using the EPS corresponding to the current value of $\tilde{n}$ by inserting $n\left(t\right)=\tilde{n}$ in Equation (4).

In the present study, the area considered is N${}_{10}^{35}$W${}_{80}^{120}$, which for $M_{\sigma }=4.0$ and $M_{\lambda }=6.0$ leads to the CDF shown in Figure 4a. When focusing on the period from 1 January 1989 to 1 January 2021, the empirical CDF comprises 218 EQ cycles and is shown in Figure 4a. In this figure, we observe that the fit

(5) $$Prob\left[n<n\left(t\right)\right]=1-\exp\left\{-{\left[\frac{n(t)}{83.46}\right]}^{0.953}\right\}$$

using the Weibull distribution provides a fair approximation with root mean square of residuals [102] equal to 0.0177. This property of the Weibull distribution is in accordance with the results found by Pasari and Sharma [79] for Himalayan EQs as well as with those later found in Reference [19] for Eastern Mediterranean.

### 2.4. Construction of Average Earthquake Potential Score Maps

Upon the selection of a date, one can estimate by means of the EQ catalog the number of ‘small’ EQs $\tilde{n}$ that occurred since the last ’large’ EQ inside a circle of radius *R* for each point in the study area. By inserting $\tilde{n}$ in Equation (5) we can obtain the corresponding EPS value. The 〈EPS〉 maps are constructed by evaluating first the value of EPS, EPS${}_{ij}$, at the points $\left(x_{ij},y_{ij}\right)$ of a ‘square’ lattice that covers the study area and then by spatially averaging these EPS values within disks of the same radius *R*:

(6) $$\langle \mathrm{EPS}\rangle \left(x,y\right)=\frac{1}{N}\sum_{i,j}^{d(x,y;x_{ij},y_{ij})\leq R}{EPS}_{ij},$$

where the summation is restricted to the lattice points whose distance $d(x,y;x_{ij},y_{ij})$ from the observation point (x,y) is smaller than or equal to *R*; *N* stands for the number of lattice points included in the sum.

In Section 3 of Reference [19], it has been shown that the 〈EPS〉 mean value $m_{n}\left(R,R^{\prime }\right)$

(7) $$m_{n}\left(R,R^{\prime }\right)=\frac{1}{N_{ij}}\sum_{ij}\langle \mathrm{EPS}\rangle \left(x_{ij},y_{ij}\right),$$

where the summation is made over all the $N_{ij}$ lattice points $\left(x_{ij},y_{ij}\right)$, estimated numerically in an 〈EPS〉 map drawn with self-consistency radius *R* and covering an area *A* of average radius $R^{\prime }(\approx$$\sqrt{A/\pi })$ may take the form

(8) $$m_{n}\left(R,R^{\prime }\right)={\left(\frac{R}{R^{\prime }}\right)}^{d_{f}},$$

where $d_{f}$ is related to [19] the fractal dimension [103] of EQ epicenters.

## 3. Results

A self-consistent method of producing 〈EPS〉 maps using a radius *R* has been suggested and applied to Eastern Mediterranean in Reference [19], as already mentioned. The date on which $\beta_{min}$ has been observed -on the basis of the analysis of the properties of ENBOSAP [21]- has been selected for drawing these 〈EPS〉 maps. The latter are produced by first estimating EPS for disks of radius *R* centered at each point of a square 0.25${}^{\circ }\times 0.25^{\circ }$ lattice covering the whole region of study and then by averaging these EPS values within the same radius *R* (see Section 2.4). A clear relation between the such made 〈EPS〉 maps and the epicenter of an impending strong EQ has been observed. Moreover, the stability of the method when changing the lattice ‘constant’ from 0.25${}^{\circ }$ to 0.10${}^{\circ }$ has been secured.

Here, we focus on the area N${}_{10}^{35}$W${}_{80}^{120}$ of intense seismicity depicted in Figure 1. For example, during the 32 year period from 1 January 1989 to 1 January 2021 we have in total 19,184 EQs with M $\geq M_{\sigma }=4.0$, i.e., approximately 50EQs/month, 28 of which have magnitude M ≥7.0, i.e., approximately 0.875 EQs/year (see Table 1). Due to this frequent occurrence of EQs of magnitude M ≥7.0, the application of the properties of ENBOSAP *k*-cores in order to identify the minima preceding these strong EQs as made in Reference [19] is not considered necessary. In other words, since on average every year we have approximately one strong EQ we just need to identify minima of the variability $\beta_{W}$ for a reasonable value of *W* as suggested by Varotsos et al. [96]. We selected W=200 corresponding to the number of EQs that occur on average every four months.

This way, the dates of the minima $\beta_{W,min}$ identified six months before each strong EQ are shown by the vertical cyan lines in Figure 2 and are also inserted in the second column of Table 1. In the case of some strong EQs, i.e., those numbered 2, 17, 18, and 27 in Table 1, a minimum of $\beta_{200}$ cannot be identified clearly before the strong EQ, thus minima of $\beta_{150}$ and/or $\beta_{100}$ have been used. The detailed behavior of the variability of the order parameter of seismicity before each strong EQ can be seen in the excerpts shown in Figure 3. An inspection of the latter figure together with Table 1 reveals that some minima may correspond to more than one strong EQ. This does not impose any actual problem, because the 〈EPS〉 maps produced for such a date can be easily compared with the epicenters of the strong EQs that followed the minimum due to the large dimensions of the study area.

**Table 1:** The dates of the variability minima $\beta_{W,min}$ for W=200 observed within 6 months before all the strong M ≥7.0 EQs in the study area N${}_{10}^{35}$W${}_{80}^{120}$ that have been selected for drawing the 〈EPS〉 maps shown in Figure 5. The lines corresponding to EQs of magnitude M ≥7.5 are indicated by typing the magnitude in bold. In the last line, the $\beta_{200,min}$, which was observed before the very recent 2021 Guerrero, Mexico EQ, is inserted (almost two weeks later the 2021 Jiquilillo, Nicaragua, M6.5 earthquake took place with an epicenter at 12.16${}^{\circ }$ N 87.85${}^{\circ }$ W).

No	$\beta_{W,\min}$ Date	EQ Date	M	Epicenter Location
1	7 March 1992	28 June 1992	7.3	34.20${}^{\circ }$ N 116.44${}^{\circ }$ W
2	17 August 1992 ${}^{a}$	2 September 1992	**7.7**	11.74${}^{\circ }$ N 87.34${}^{\circ }$ W
3	18 May 1993	10 September 1993	7.2	14.72${}^{\circ }$ N 92.64${}^{\circ }$ W
4	2 September 1995	14 September 1995	7.4	16.78${}^{\circ }$ N 98.60${}^{\circ }$ W
5	2 September 1995	9 October 1995	**8.0**	19.05${}^{\circ }$ N 104.20${}^{\circ }$ W
6	2 September 1995	21 October 1995	7.2	16.84${}^{\circ }$ N 93.47${}^{\circ }$ W
7	25 January 1996	25 February 1996	7.1	15.98${}^{\circ }$ N 98.07${}^{\circ }$ W
8	15 July 1996	11 January 1997	7.2	18.22${}^{\circ }$ N 102.76${}^{\circ }$ W
9	3 April 1999	15 June 1999	7.0	18.39${}^{\circ }$ N 97.44${}^{\circ }$ W
10	3 April 1999	30 September 1999	**7.5**	16.06${}^{\circ }$ N 96.93${}^{\circ }$ W
11	3 April 1999	16 October 1999	7.1	34.59${}^{\circ }$ N 116.27${}^{\circ }$ W
12	19 December 2000	13 January 2001	**7.7**	13.05${}^{\circ }$ N 88.66${}^{\circ }$ W
13	1 August 2002	22 January 2003	**7.6**	18.77${}^{\circ }$ N 104.10${}^{\circ }$ W
14	9 April 2004	9 October 2004	7.0	11.42${}^{\circ }$ N 86.67${}^{\circ }$ W
15	27 April 2009	28 May 2009	7.3	16.73${}^{\circ }$ N 86.22${}^{\circ }$ W
16	12 March 2010	4 April 2010	7.2	32.30${}^{\circ }$ N 115.28${}^{\circ }$ W
17	13 March 2012 ${}^{a}$	20 March 2012	7.4	16.49${}^{\circ }$ N 98.23${}^{\circ }$ W
18	6 April 2012 ${}^{b}$	12 April 2012	7.0	28.70${}^{\circ }$ N 113.10${}^{\circ }$ W
19	1 July 2012	27 August 2012	7.3	12.14${}^{\circ }$ N 88.59${}^{\circ }$ W
20	1 July 2012	5 September 2012	**7.6**	10.09${}^{\circ }$ N 85.31${}^{\circ }$ W
21	4 November 2012	7 November 2012	7.4	13.99${}^{\circ }$ N 91.89${}^{\circ }$ W
22	27 February 2014	18 April 2014	7.2	17.40${}^{\circ }$ N 100.97${}^{\circ }$ W
23	8 October 2014	14 October 2014	7.3	12.53${}^{\circ }$ N 88.12${}^{\circ }$ W
24	11 May 2017	8 September 2017	**8.2**	15.02${}^{\circ }$ N 93.90${}^{\circ }$ W
25	11 September 2017	19 September 2017	7.1	18.55${}^{\circ }$ N 98.49${}^{\circ }$ W
26	14 December 2017	10 January 2018	**7.5**	17.48${}^{\circ }$ N 83.52${}^{\circ }$ W
27	10 January 2018 ${}^{a,b}$	16 February 2018	7.2	16.39${}^{\circ }$ N 97.98${}^{\circ }$ W
28	16 April 2020	23 June 2020	7.4	15.89${}^{\circ }$ N 96.01${}^{\circ }$ W
29	10 June 2021	8 September 2021	7.0	16.98${}^{\circ }$ N 99.77${}^{\circ }$ W

${}^{a}$ This date comes from W=100. ${}^{b}$ This date comes from W=150.

**Figure 5:**
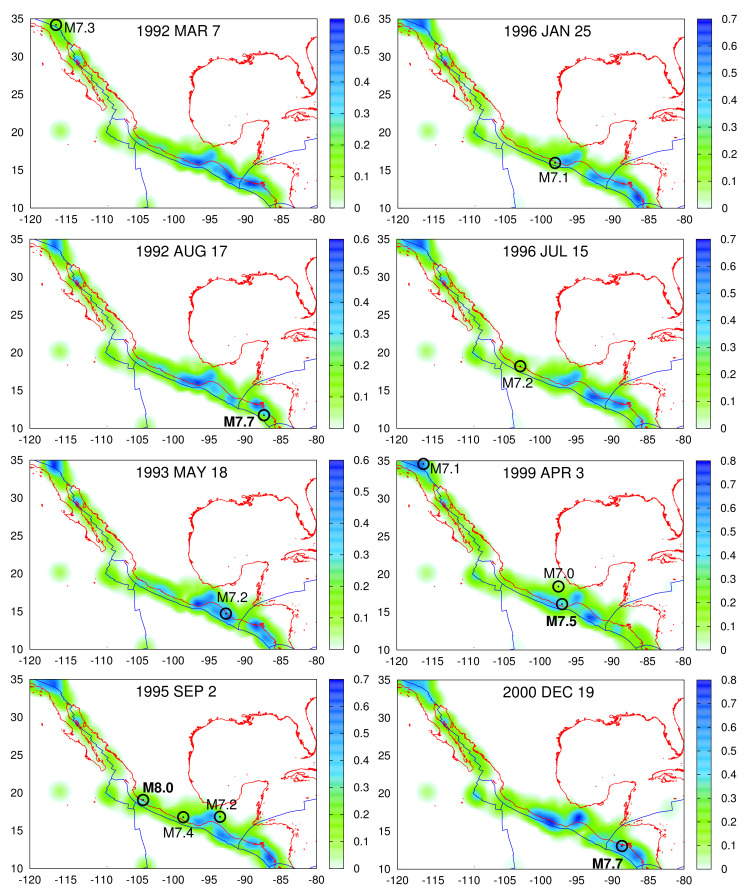

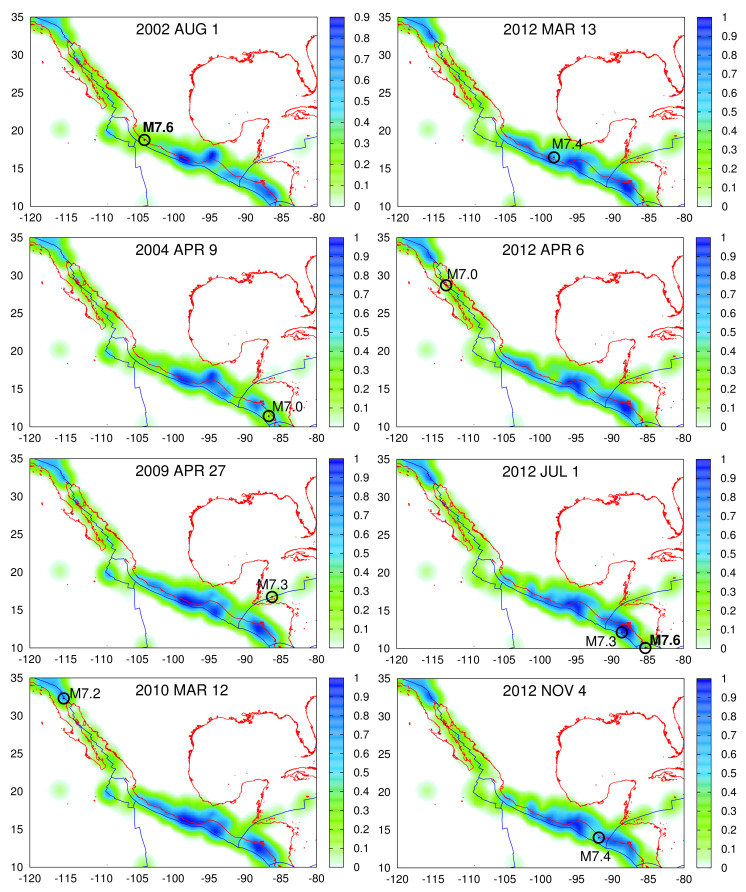

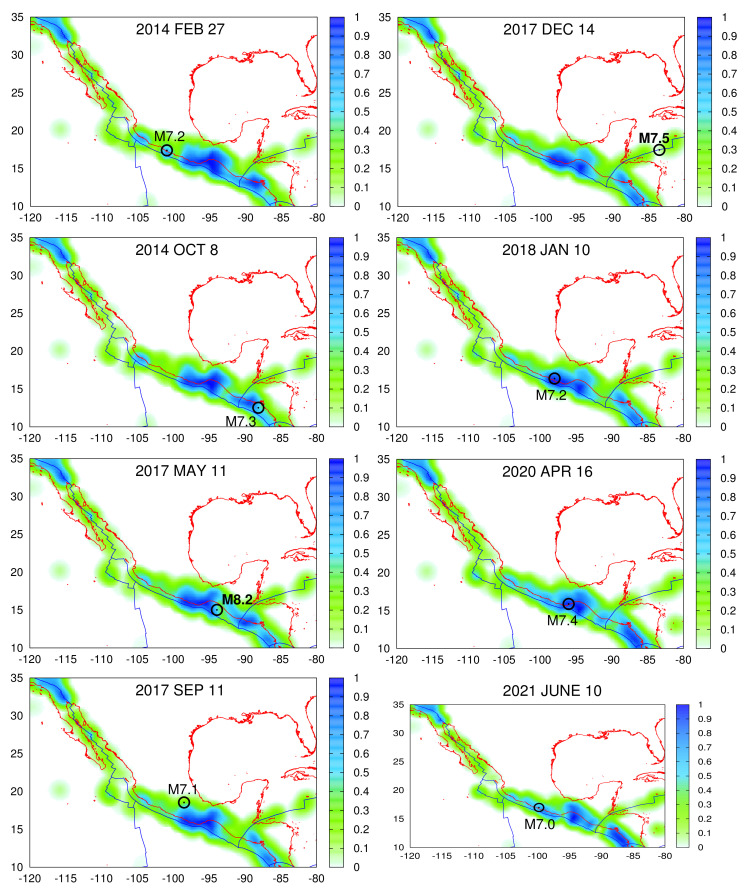
Maps of the study area N${}_{10}^{35}$W${}_{80}^{120}$ together with the plate boundaries (blue) according to Reference [73] depicting by color scale the 〈EPS〉 for *R* = 100 km at the dates of $\beta_{W,min}$ inserted in Table 1. The EQ epicenters are shown by the (black) open circles in each case and their magnitude is typed boldface when M≥7.5.

In Figure 5, using a 0.2${}^{\circ }\times 0.2^{\circ }$ grid, we depict the 〈EPS〉 maps determined for *R* = 100 km for each $\beta_{W,min}$ date of Table 1 together with the location of the epicenters of the strong EQs that followed within the next six months. We observe that the EQ epicenters compare favorably with the contours of 〈EPS〉. This relation will be further elaborated in the next section.

## 4. Discussion

**Figure 6:**
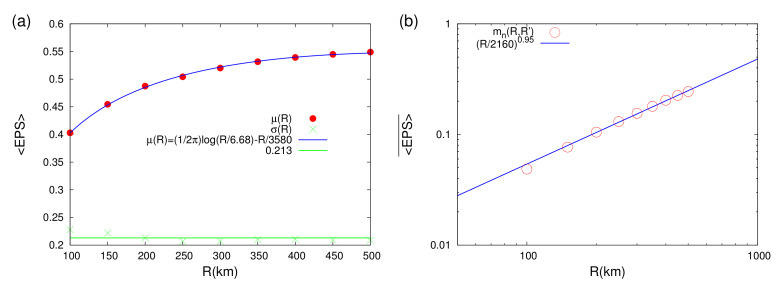
Dependence of the 〈EPS〉 statistics on the coarse grain radius *R*: Panel (**a**) depicts the average value μ(R) (red bullets) and the STD σ(R) (green crosses) of the 〈EPS〉 values closest to each epicenter of the first 28 EQs of Table 1. The fitting function μ(R) (blue) and the average value of σ(R) (green horizontal line) are also shown. Panel (**b**) depicts the mean value $\bar{\langle \mathrm{EPS}\rangle }$ over all the grid points of the 〈EPS〉 maps shown in all but the last panel of Figure 5. This mean value labeled $m_{n}\left(R,R^{\prime }\right)$ (open red circles) is plotted vs. *R* together with the corresponding power law fit (blue straight line).

We first studied the statistics of the 〈EPS〉 values closest to the epicenters of the strong EQs with M ≥7.0 during the period 1 January 1989 to 1 January 2021, i.e., the first 28 EQs of Table 1. The results for the mean value μ(R) and the standard deviation (STD) σ(R) are plotted versus *R* in Figure 6a, which indicates a characteristic behavior of a monotonically increasing μ(R). A further inspection of Figure 6a reveals that it is compatible with the functional form

(9) $$\mu \left(R\right)=\frac{1}{2\pi }\log\left[\frac{R}{6.68(6)}\right]-\frac{R}{3580(50)}$$

which includes the characteristic logarithmic singular part of the Green’s function in two dimensions, see, e.g., Equation (9.96c) in paragraph 3 of Section 9.2.2.3 of Bronshtein et al. [104], together with a linear (non-singular) correction term. The presence of this singular logarithmic part when analyzing 〈EPS〉 closest to the strong EQ epicenters should be considered as a strong indication of the interrelation between the location of the epicenters and the corresponding 〈EPS〉 maps. It signifies that the future epicenters seem to act similar to sources in the two dimensional 〈EPS〉 field.

The above point is further strengthened by the fact that when considering the 〈EPS〉 mean value $\bar{\langle \mathrm{EPS}\rangle }$ over all the lattice points of the grid (square lattice) for all the 〈EPS〉 maps of Figure 5 that correspond to the first 28 EQs of Table 1 the singular behavior disappears, see Figure 6b. The functional form observed in Figure 6b indicates a value of $d_{f}$ = 0.95(2) which is compatible with the almost one-dimensional fault structure of Figure 1 (cf. the latter is dominated by the faults along the Pacific coast). Moreover, a comparison of Figure 6b with Equation (8) reveals that $R^{\prime }$ = 2160(80) km, which compares favorably with the quantity $\sqrt{A/\pi }\approx$ 2000 km when considering the study area *A* of 25${}^{\circ }\times 40^{\circ }$.

**Figure 7:**
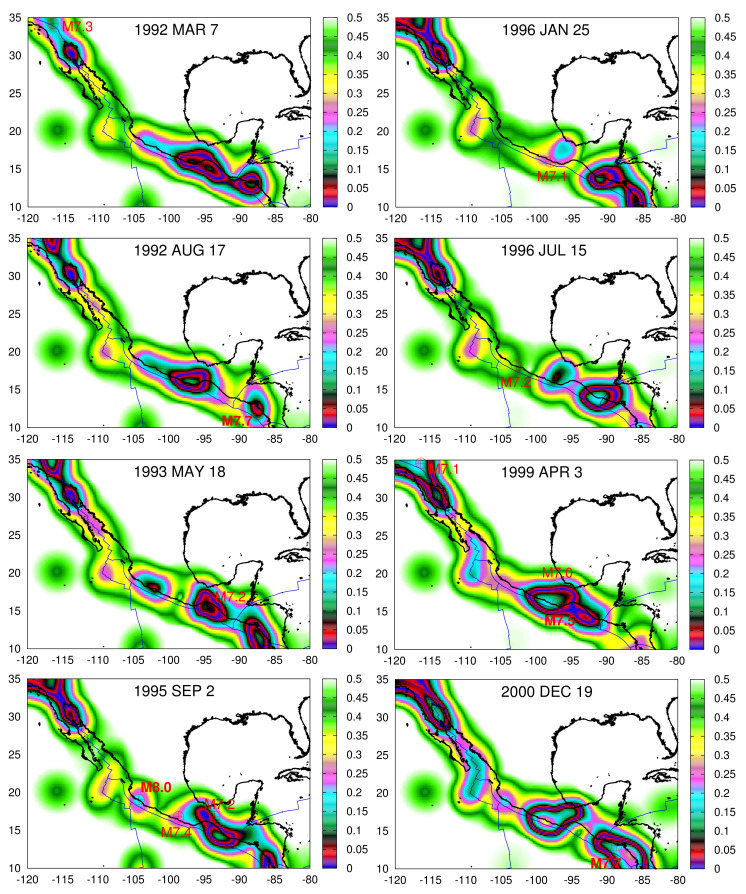

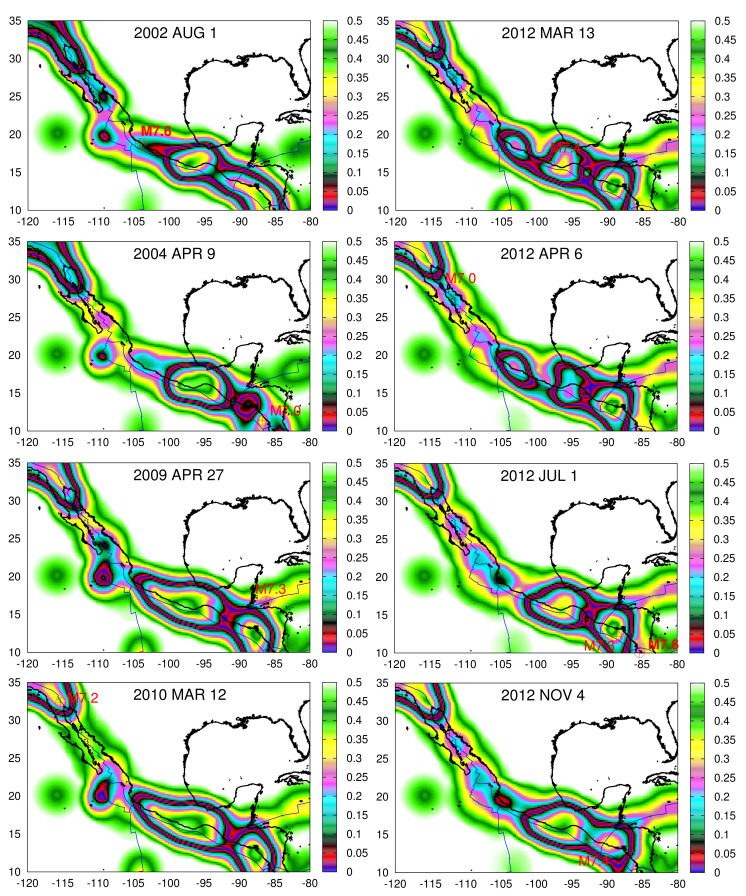

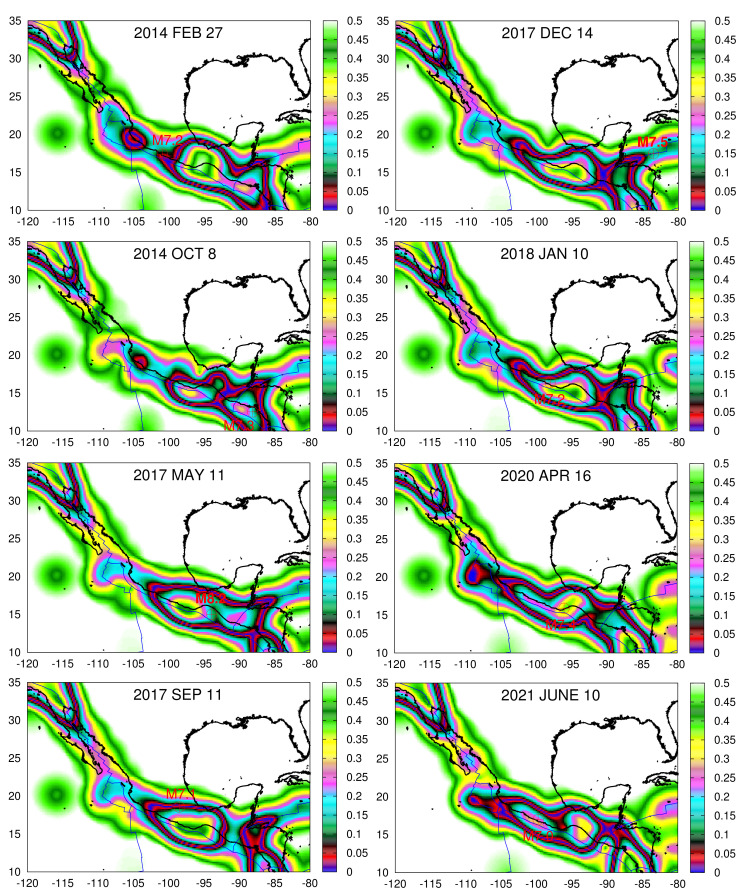
Maps of the study area N${}_{10}^{35}$W${}_{80}^{120}$ together with the plate boundaries (blue) according to Bird [73] depicting by color scale the quantity |〈EPS〉−0.5| for *R* = 200 km at the dates of $\beta_{W,min}$ inserted in Table 1. The EQ epicenters are shown by the (red) circles with pluses in each case and their magnitude is typed boldface when M≥7.5.

Furthermore, we studied the statistics of 〈EPS〉 values closest to the epicenters of the 8 EQs with M≥7.5 (shown with bold letters in Table 1 and Figure 5). It was found that for R=250 km, a particularly interesting behavior is observed: Namely a bimodal distribution appears with mean value 0.55 and two lobes ±0.15 from the mean. This, in conjunction with the fact that at R=250 km, Figure 6a reveals an average value of 0.50, encouraged us to investigate the maps of |〈EPS〉−0.5| for values of *R* around 250 km (cf. in previous studies [19,58] R=250 km has also been found of particular importance). Such a study revealed that |〈EPS〉−0.5| maps may be useful and the optimum results (shown in Figure 7) were obtained when using R=200 km.

An inspection of Figure 7 reveals that if we ignore the case of the 1997 Michoacan M7.2 EQ [105] that corresponds to the $\beta_{200,min}$ observed on 15 July 1996, the epicenters of all the other 28 strong EQs of magnitude M ≥7.0 lie inside or up to 120 km away from the region shaded with tones of cyan color, i.e., with |〈EPS〉−0.5|∈[0.1,0.24]. This latter region on average covers only 8.5% of the total area with a STD of 2.3%. Additionally, 25 out of the 29 strong EQ epicenters of Table 1 lie within 65km away from the same region (cf. the 1993 M7.2, the 2012 M7.3, and the 2020 M7.4 EQs lie away 118 km, 87 km, and 115 km, respectively).

The results shown in Figure 7 do not, of course, solve the very difficult problem of finding the epicenter of a future strong EQ since the areas covered by the tones of cyan color are spatially distributed in a rather large area, but clearly indicate that 〈EPS〉 maps definitely include information concerning the epicenter of a future strong EQ. Our efforts to improve these results are in progress by applying this method to other seismically active areas around the globe (cf. the operation of SES measuring stations [106] is an essential factor for such an improvement since additional information on the future epicentral area can be thus obtained).

## 5. Conclusions

Here, we studied the variability $\beta_{W}$ of the order parameter of seismicity $\kappa_{1}$ (introduced by natural time analysis) together with earthquake nowcasting within the highly active seismic region N${}_{10}^{35}$W${}_{80}^{120}$ that covers Southern California, Mexico, and part of Central America. We suggest a self-consistent method of constructing 〈EPS〉 maps to obtain an estimation of the epicenter location of a future strong EQ of magnitude M ≥7.0 in this region. The study of 〈EPS〉 values closest to the strong EQ epicenters showed a logarithmic behavior, which is reminiscent of the Green’s function in two dimensions. This is compatible with the view that the future epicenters act like ‘sources’ in these two dimensional maps. Using NTA and EN the epicenter of a future strong M ≥7.0 EQ was estimated to lie in the vicinity of a region covering on average only 8.5% of the total study area with a hit rate 28/29(≈96.5%).

## Data Availability

Earthquake data come from the United States National Earthquake Information Center PDE and were downloaded from the United States Geological Survey, Earthquake HazardsProgram [84]. The last date the data were accessed was 21 September 2021. Gnuplot [107] was used for the preparation of the figures. The coast lines were imported from GEODAS Coastline Extractor [108]. The datasets generated during and/or analyzed during the current study are available from the corresponding author on reasonable request.

## Abbreviations

The following abbreviations are used in this manuscript:

CDF	Cumulative distribution function
EN	Earthquake nowcasting
ENBOSAP	Earthquake networks based on similar activity patterns
EPS	Earthquake potential score
EQ	Earthquake
M	magnitude
NEIC	National earthquake information center
NTA	Natural time analysis
PDE	Preliminary determination of epicenters
SES	Seismic electric signals
STD	Standard deviation
USGS	United States geological survey
